# Fibroblast growth factor 21 in breast milk controls neonatal intestine function

**DOI:** 10.1038/srep13717

**Published:** 2015-09-02

**Authors:** Aleix Gavaldà-Navarro, Elayne Hondares, Marta Giralt, Teresa Mampel, Roser Iglesias, Francesc Villarroya

**Affiliations:** 1 Departament de Bioquimica i Biologia Molecular, Institute of Biomedicine (IBUB), University of Barcelona, Av Diagonal 643, 08028 Barcelona, Catalonia, Spain; 2 CIBER Fisiopatologia de la Obesidad y Nutrición, Av Diagonal 643, 08028 Barcelona, Catalonia, Spain

## Abstract

FGF21 is a hormonal factor with important functions in the control of metabolism. FGF21 is found in rodent and human milk. Radiolabeled FGF21 administered to lactating dams accumulates in milk and is transferred to neonatal gut. The small intestine of neonatal (but not adult) mice highly expresses β-Klotho in the luminal area. FGF21-KO pups fed by FGF21-KO dams showed decreased expression and circulating levels of incretins (GIP and GLP-1), reduced gene expression of intestinal lactase and maltase-glucoamylase, and low levels of galactose in plasma, all associated with a mild decrease in body weight. When FGF21-KO pups were nursed by wild-type dams (expressing FGF21 in milk), intestinal peptides and digestive enzymes were up-regulated, lactase enzymatic activity was induced, and galactose levels and body weight were normalized. Neonatal intestine explants were sensitive to FGF21, as evidenced by enhanced ERK1/2 phosphorylation. Oral infusion of FGF21 into neonatal pups induced expression of intestinal hormone factors and digestive enzymes, lactase activity and lactose absorption. These findings reveal a novel role of FGF21 as a hormonal factor contributing to neonatal intestinal function via its presence in maternal milk. Appropriate signaling of FGF21 to neonate is necessary to ensure optimal digestive and endocrine function in developing intestine.

Milk, which is the essential source of nutrition for mammalian neonates provides major nutrients, vitamins, minerals and water, as well as numerous biologically active molecules, including growth factors, hormones and adipokines^1^,^2^,^3^. These biological active factors play important roles in maintaining neonatal homeostasis and supporting proper neonatal development. Some of these factors, such as leptin and adiponectin, are absorbed from the lumen of the gut into the neonatal circulation^4^. Leptin in breast milk is known to be involved in the control of energy balance during the post-natal period^5^. Other factors present in milk are believed to function in protecting the gut; for example epidermal growth factor present in milk facilitates the development of essential digestive functions and regulates the maturation of the gastrointestinal tract^2^,^6^.

The identification of the biologically active factors in milk should help improve our understanding and management of optimal nutrition strategies for childhood development. The enteral nutrition of intestinally immature neonates relies on breast milk because of unique properties and composition of milk^7^. Nutrition in early childhood has been implicated in the long-term tendencies towards obesity and metabolic syndrome, and epidemiological data indicate that breastfeeding protects against such metabolic alterations in later life^3^. It is considered that these protective effects may involve the effects of bioactive compounds found in breast milk.

Fibroblast growth factor 21 (FGF21) is a hormonal factor that plays important roles in controlling glucose homeostasis and energy metabolism^8^. In adults, FGF21 is produced mainly in the liver; it favors glucose uptake by peripheral tissues, and thus possesses anti-diabetic properties^9^. FGF21 is also expressed in white adipose tissue (WAT), where it has been proposed to play autocrine effects^10^, and in brown adipose tissue (BAT), which releases FGF21 after thermogenic activation^11^. A strong regulation of the FGF21 system has been reported in mouse neonates: FGF21 levels are very low in fetuses, but the blood levels of FGF21 rise dramatically after birth and the initiation of suckling; this is associated with the induction of hepatic FGF21 gene expression in response to fatty acids contained in milk. FGF21 has powerful effects in neonates, including the induction of BAT thermogenesis, which is a key process in the adaptation of neonates to the extrauterine environment^12^.

In the present study, we identified the presence of substantial amounts of FGF21 in milk, show that milk FGF21 is transferred to the neonatal gut, and report that milk FGF21 supports intestinal function in the neonate.

## Results

### FGF21 is present in milk

**Figure 1:**
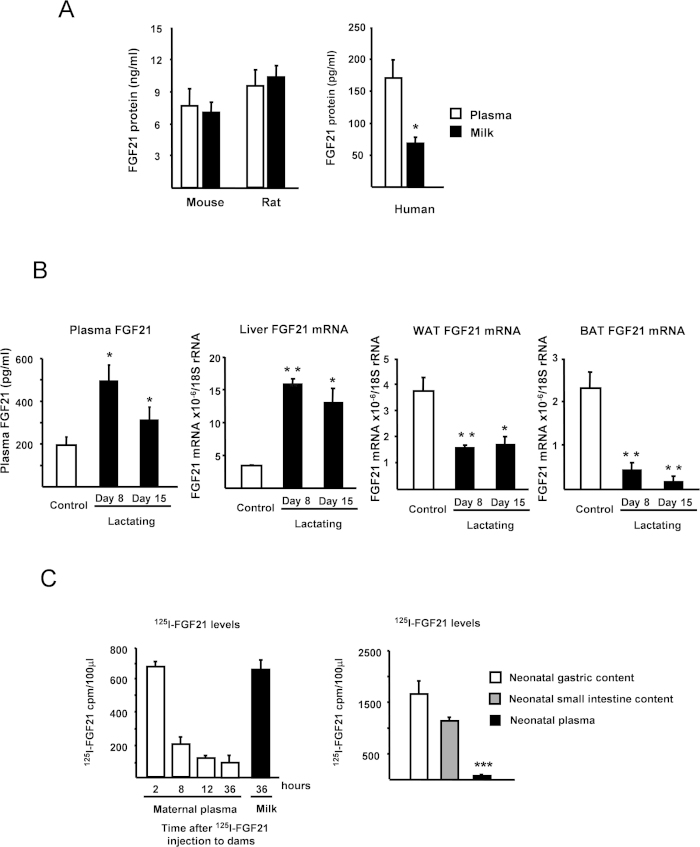
FGF21 is present in milk and transferred to neonatal gut. (**A**) FGF21 protein levels in plasma (P) and milk (M) from mice, rats and humans. Data are means ± SEM of 6 samples in rats, 8 samples in mice and 20 samples in humans. (**B**) Plasma FGF21 levels and FGF21 mRNA expression in liver, WAT and BAT from female mice at days 8 and 15 of lactation, and non-lactating female controls. Statistically significant changes versus controls are shown as *P < 0.05, **P < 0.01. (**C**) Radioactivity tracing of ^125^I-FGF21 in plasma and milk of lactating dams at the indicated hours after i.p. administration of ^125^I-FGF21 to dams. Data are means ± SEM of TCA-precipitated material from 4 independent assays. (**D**) Radioactivity tracing of ^125^I-FGF21 along the stomach and small intestine of 15 day-old pups fed by ^125^I-FGF21-treated lactating dams for 36 h after treatment of the dams. Data are means ± SEM of TCA-precipitated material from 4 independent assays. Statistically significant differences relative to plasma levels are shown as *P < 0.001.

We analyzed the presence of FGF21 in human, rat and mouse milk and plasma samples using specific ELISA assays. As shown in Fig. 1A, FGF21 was detected in maternal milk at concentrations that varied by species. Similar FGF21 levels were present in plasma and milk from lactating mice and rats, whereas the FGF21 levels in human milk were almost half those found in the corresponding plasma. The FGF21 levels in human milk did not differ significantly when colostrum and milk at different stages of lactation were measured (Supplemental Figure 1).

Since the mammary gland is responsible for the production of some of the hormones present in breast milk [e.g leptin]^13^, we analyzed FGF21 mRNA expression in mammary gland from lactating dams. However, FGF21 mRNA expression was undetectable in mammary glands from mice at distinct stages of lactation (days 4, 8, 15 and 21 of lactation) as well as in resting mammary gland from non-lactating mice (Supplemental Table 1). Similarly, neither RNA from resting human mammary gland nor RNA from cells obtained from breastmilk, representative of mammary epithelium, showed detectable FGF21 mRNA expression (Supplemental Table 2), suggesting that direct production of FGF21 by mammary gland is unlikely to be responsible for the presence of FGF21 in milk. We then determined the plasma FGF21 levels of lactating mice, and observed that plasma FGF21 levels were higher on days 8 and 15 of lactation, compared to control, non-lactating, female mice (Fig. 1B). We further analyzed the gene expression of FGF21 in the main FGF21-expressing tissues from lactating dams, and found that FGF21 mRNA levels were down-regulated in WAT and BAT but markedly induced in the liver in lactating females compared to control females (Fig. 1B). These results suggest that changes in liver FGF21 expression are the main determinants of the high FGF21 levels observed in lactating mice.

### FGF21 is transferred from blood to milk in lactating dams and subsequently to neonatal gut in suckling pups

To test whether the FGF21 observed in maternal plasma passes to milk, lactating dams were intraperitoneally injected with ^125^I-FGF21, and the presence of ^125^I-FGF21 was monitored in plasma and milk (Fig. 1C, left). The level of ^125^I-FGF21 in serum peaked 2 h after injection and declined thereafter. Thirty-six hours after the administration of ^125^I-FGF21 to lactating dams, high levels of ^125^I-FGF21 were observed in milk (Fig. 1C, left). At this point, milk ^125^I-FGF21 levels were in the range of the high levels attained in plasma just 2 h after injection. These results support the notion that FGF21 is transferred from the blood of lactating dams to their milk. We also studied the presence of ^125^I-FGF21 in the stomach, gut and plasma of suckling neonates. As shown in Fig. 1C (right), ^125^I-FGF21 was detected in gastric and intestinal contents of suckling mice after 36 h of milk intake from ^125^I-FGF21-treated mothers. When we resolved samples of the gut content by electrophoresis and subjected them to autoradiography, our results confirmed the presence of the 23 kDa band corresponding to bona-fide ^125^I-FGF21 (Supplemental Figure 2). In contrast, the levels of ^125^I-FGF21 in the serum samples of neonates were very low (less than 5% of the stomach cpm values), indicating that the milk FGF21 is mainly retained in the stomach and intestine of the neonate, and is not significantly transferred to the systemic circulation. This suggests that FGF21 is not contributing to the circulating pool of FGF21 in the neonate and, if having a biological effect, it is likely to occur locally in the gut.

### β-Klotho is highly expressed in the luminal area of the small intestine in neonates but not in adults

**Figure 2:**
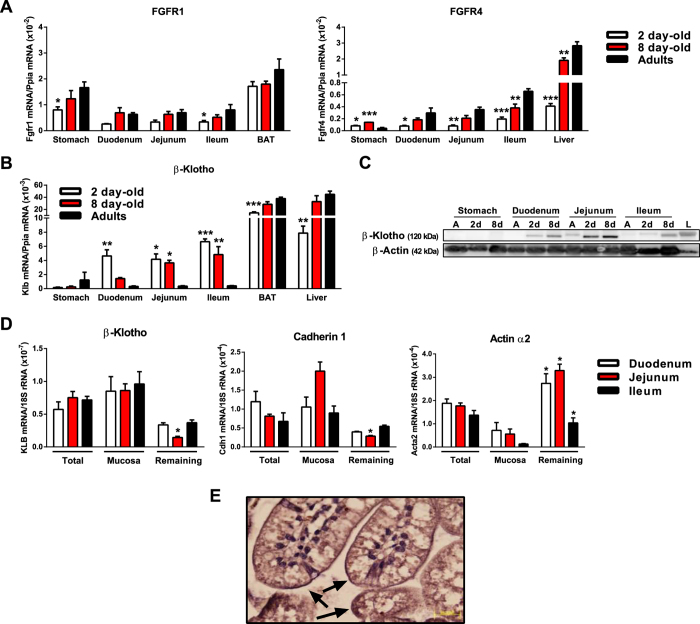
β-Klotho is highly expressed in the luminal area of the neonatal small intestine in mice. (**A**) FGFR1 and FGFR4 mRNA levels in stomach, duodenum, jejunum, ileum and BAT or liver from 2- and 8-day-old pups and adults. (**B**) β-Klotho mRNA levels in the stomach, duodenum, jejunum, ileum, BAT and liver from 2- and 8-day-old pups and adults. (**C**) Representative immunoblot analysis of β-Klotho protein levels in the stomach, duodenum, jejunum, ileum (40 μg protein/lane) from 2-day-old (2d) and 8-day-old (8d) pups and liver (L; 15 μg protein/lane). (**D**) mRNAs expression for β-Klotho, cadherin-1 and actin alpha-2 in total small intestine, scrapped intestinal mucosa and the remaining tissue in 15-day old neonates. (**E**) Immunohistochemical analysis of β-Klotho protein levels in ileum sections from 8 day-old neonates. Bars are means ± SEM of 4–6 independent samples per group. Statistically significant changes relative to adults (in A and B), or in mucosa versus remaining tissue for each intestine section (in D), are shown as *P < 0.05, **P < 0.01 and ***P < 0.001.

Considering that neonatal gastro-intestinal system may be locally exposed to milk FGF21, we analyzed the expression of FGF receptors (FGFRs; which mediate the effects of FGF21) in the stomach and small intestine during early postnatal development. We also determined the expression of β-Klotho, which is the auxiliary co-receptor required for specific response of cells and tissues to FGF21. The mRNA expression levels of FGFR1 in adult mice were somewhat lower in the stomach and intestine than in BAT, and those in the neonatal stomach and small intestine were similar to that in the adult small intestine (Fig. 2A, left). For FGFR4, the transcript levels in adult mice were lower in stomach and intestine compared to the liver; and those in the neonatal stomach and intestine were somewhat lower than those in the corresponding adult tissues (Fig. 2A, right). The mRNA expression levels of FGFR2 and FGFR3 in the neonatal stomach and small intestine were lower than that in the adult small intestine (Supplemental Figure 3).

In adults, the mRNA levels of β-Klotho were much lower in the stomach and intestine compared to BAT or liver (Fig. 2B); this is consistent with the current belief that the adult gastro-intestinal system is not an important target of FGF21^14^. However, the mRNA expression of β-Klotho was markedly high in the neonatal small intestine at both day 2 and day 8 of age. It was also high in all three sections of the small intestine, and was around 50–60-fold higher in the neonatal jejunum and ileum compared to the corresponding adult tissues (Fig. 2B).

Immunoblot assays showed that the β-Klotho protein was substantially expressed in distinct sections of the neonatal small intestine, but not in the adult small intestine (Fig. 2C). The levels of β-Klotho mRNA were compared between mucosal samples scraped from the neonatal small intestine and the remaining intestinal tissue. Our results indicated that β-Klotho was preferentially expressed in the neonatal intestinal mucosa, in parallel with cadherin-1 (a marker of the intestinal epithelium) but in opposition to actin α-2 (a marker of smooth muscle in the intestine) (Fig. 2D). We used immunocytochemistry to assess the preferential site(s) of β-Klotho expression, and observed an β-Klotho immunoreactive signal in the luminal surface of the neonatal small intestine (Fig. 2E).

Together, our data indicate that β-Klotho is specifically expressed at high levels in the small intestine epithelia of neonatal mice, suggesting that the neonatal small intestine may be highly responsive to FGF21.

### The levels of incretins and digestive enzymes and blood galactose levels are decreased in FGF21-KO neonates fed by FGF21-KO dams. Feeding neonates by wild-type dams prevents intestinal alterations

**Figure 3:**
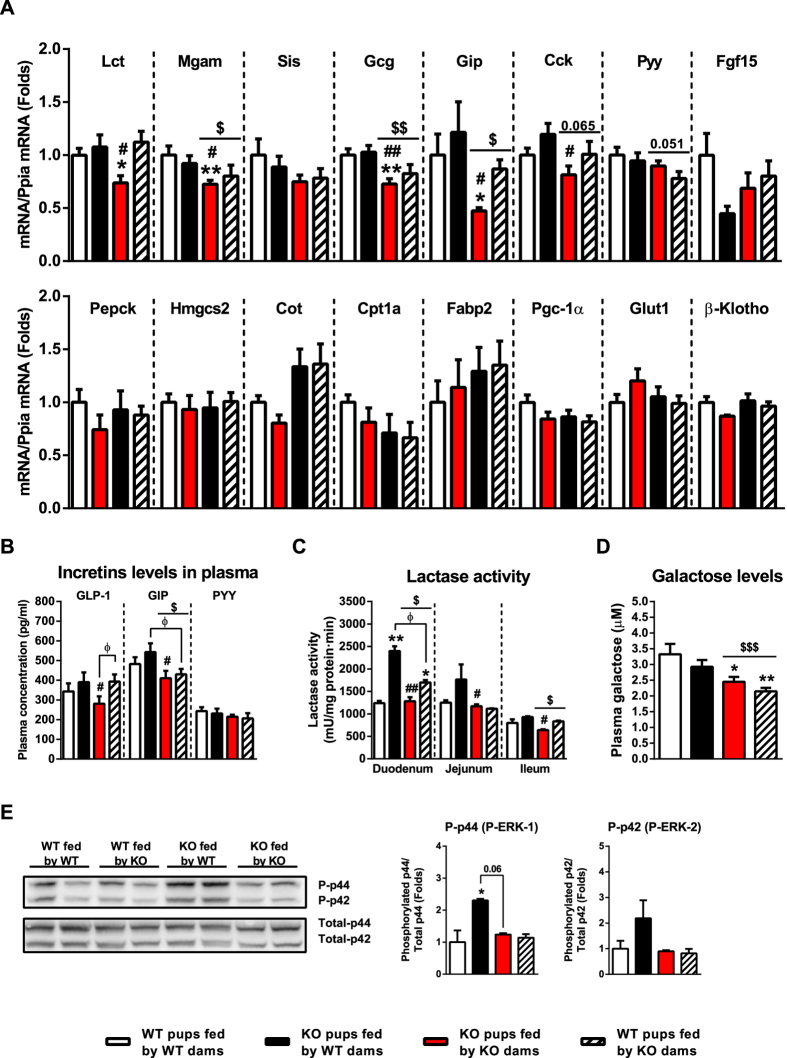
Effects of FGF21-KO lactation on the neonatal intestine. Data are shown from wild-type (WT) pups fed by WT dams, FGF21-KO pups fed by FGF21-KO dams, FGF21-KO pups fed by WT dams and FGF21-WT pups fed by KO dams, from day 1 after birth to postnatal day 8. (**A**) Transcript levels in the ileum. (**B**) Plasma levels of GLP-1, GIP and PYY. (**C**) Lactase activity in the ileum. (**D**) Plasma levels of galactose. (**E**) Levels of ERK1/2 phosphorylation (representative immunoblot, 40 μg protein/lane, left; quantification of the immunoblot signal intensity, right). Bars are means ± SEM of 5 independent samples per group, each one being pooled samples from 4–5 independent litters. Statistically significant changes versus WT pups fed by WT dams are shown as *P < 0.05, **P < 0.01 and those between FGF21-KO pups fed by WT and those fed by FGF21-KO dams are shown as ^#^P < 0.05, ^##^P < 0.01. Statistically significant changes between WT pups fed by KO dams and other groups are shown as ^Φ^P < 0.05. Statistically significant changes between pups fed by WT dams and KO dams, retrieved by two-factor ANOVA analysis, are shown as ^$^P < 0.05, ^$$^P < 0.01 and ^$$$^P < 0.001.

To gain insight into the role of milk FGF21 in the neonatal intestine, we analyzed FGF21-knockout (KO) pups nursed by FGF21-KO dams (whose milk lacks FGF21) or by wild-type (WT) dams (whose milk contains FGF21), and compared them with WT pups nursed by WT dams or nursed by FGF21-KO dams. FGF21-KO pups fed by FGF21-KO dams showed significantly lower expression levels of the genes encoding the digestive enzymes lactase (Lct) and maltase glucoamylase (Mgam) in the ileum. A similar (but non-significant) trend was observed for sucrase isomaltase (Sis). We also observed reductions in the expression levels of several genes that encode intestinal hormone factors in the ileum, namely the proglucacon gene (Gcg, encoding glucagon-like peptide-1, GLP-1, in intestine), the glucose-inhibitory peptide-encoding (Gip) gene, and the cholecystokinin (Cck) gene, but not the peptide YY (Pyy) or FGF15 genes. When WT pups fed by FGF21-KO dams were analyzed a similar trend was observed, with a reduction in Mgam, Gcg and Gip levels. Multivariate analysis of the whole four-group experimental population indicated that the nursing genotype (i.e. presence/absence of FGF21 in milk) determines significantly low expression of these genes. FGF21-KO pups fed by WT dams showed no such down-regulation of genes encoding intestinal digestive enzymes and intestinal hormone factors (Fig. 3A, top).

The expression levels of several metabolic genes encoding key proteins involved in glucose and lipid metabolism, which are known to be highly expressed in the neonatal (but not adult) small intestine^15^,^16^ were unaffected in FGF21-KO pups or WT pups regardless of whether they were fed by FGF21-KO or WT dams (Fig. 3A, bottom). Furthermore, none of the gene expression changes found in the small intestine of neonatal FGF21-KO mice nursed by FGF21-KO dams were observed in the intestine of FGF21-KO adult mice (Supplemental Figure 4).

Consistent with the above findings, the circulating levels of the incretins, GLP-1 and GIP, tended to be lowered in FGF21-KO neonates, but were rescued when these pups were fed by WT dams (Fig. 3B). In WT pups fed FGF21-KO dams only GIP levels were similarly lowered. No significant reduction was observed in the lactase activity of FGF21-KO pups fed by FGF21-KO. However, feeding FGF21-KO pups with FGF21-containing milk induced the lactase activity in the small intestine. Multivariate analysis revealed that the nursing dam’s genotype is associated to statistically significant reduction in lactase activity in duodenum and jejunum (Fig. 3C).

To estimate the consequences of intestine changes on lactose digestion performance we analyzed galactose levels in pups’ blood. Results indicated a significant reduction in galactose levels in FGF21-KO pups fed by dams lacking FGF21 in milk either in WT pups or in FGF21-KO pups (Fig. 3D).

To further examine the potential action of milk FGF21 on the neonatal small intestine, we determined ERK1/2 phosphorylation levels in our experimental setting. FGF21-KO pups fed WT (FGF21-containing) milk showed a significant induction of ERK1/2 phosphorylation compared to FGF21-KO pups fed by FGF21-KO dams (Fig. 3E), suggesting that FGF21 in milk enhances ERK1/2 phosphorylation.

Based on these results, we conclude that feeding FGF21-KO pups with FGF21-lacking milk alters the pups’ intestinal enzyme and hormonal profile, but that this can be prevented by feeding KO pups with FGF21-containing milk. WT pups fed milk lacking FGF21 show similar, although somewhat more moderate, alterations. Our characterization of the nutritional, biochemical and hormonal compositions of the milk from FGF21-KO dams did not reveal any major difference relative to WT milk (Supplemental Table 3). We therefore propose that the FGF21 present in milk from WT dams may act on the neonatal intestine, thereby preventing the alterations observed in FGF21-KO pups.

**Figure 4:**
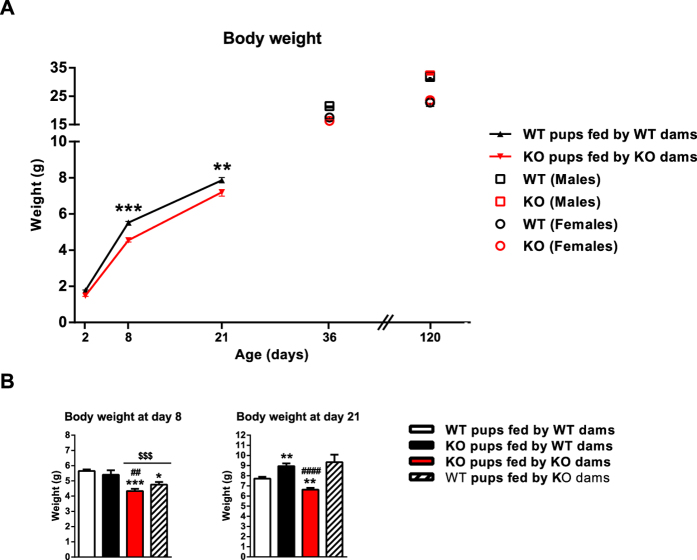
Body weight of FGF21-KO mice nursed by FGF-KO dams and WT mice nursed by WT dams and effects of nursing of FGF21-KO mice by WT dams on neonatal body weight. (**A**) Weights of neonatal WT pups nursed by WT dams and FGF21-KO pups nursed by FGF21-KO dams were registered periodically during 4 months. (**B**) Weights of WT pups nursed by WT or by KO dams and FGF21-KO pups nursed by FGF21-KO or by WT dams were registered at days 8 and 21. Results are the means + SEM from 5–6 independent litters. For adults, data are means of 10 individuals from, at least, 2 independent litters. Statistically significant changes versus WT pups fed by WT dams are shown as *P < 0.05, **P < 0.01 and those between FGF21-KO pups fed by WT and those fed by FGF21-KO dams are shown as ^#^P < 0.05, ^##^P < 0.01. Statistically significant changes between pups fed by WT dams and KO dams, retrieved by two-factor ANOVA analysis, are shown as ^$^P < 0.05, ^$$^P < 0.01 and ^$$$^P < 0.001.

The physiological, biochemical and hormonal parameters of neonates were determined under distinct maternal nursing conditions. We found that FGF21-KO pups delivered by FGF21-KO dams did not differ in body weight at birth relative to WT pups. As lactation proceeded, however, FGF21-KO pups nursed by FGF21-KO dams showed moderate (but significant), reductions in body weight compared to WT pups (Fig. 4A). This was a transient phenomenon, as FGF21-KO mice that had been nursed by FGF21-KO dams did not show significant differences in body weight relative to controls after weaning, neither males nor females. FGF21-KO pups nursed by WT dams did not show this neonatal reduction in body weight (Fig. 4B). When WT pups were nursed by dams lacking FGF21 in milk, there was also a transient reduction in body weight, which, in this case, was already normalized at day 21 after birth.

Assessment of biochemical parameters in this experimental setting didn’t reveal significant changes in single group-based comparisons. However, multivariate analysis indicated that glucose and triglyceride levels were significantly reduced in association with nursing dam’s genotype but not with pup’s genotype (Supplemental Table 4).

### FGF21 induces the gene expression of intestinal hormones and digestive enzymes in neonatal intestine explants

**Figure 5:**
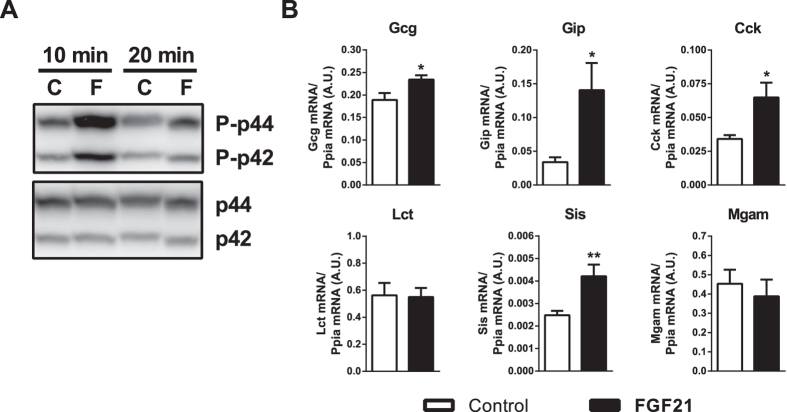
FGF21 induces the gene expression of intestinal hormones and digestive enzymes in neonatal intestine explants. (**A**) Eight-day-old mouse small intestine explants were treated with 10 nM FGF21 for 10 min and 20 min, and the levels of phosphorylated ERK1/2 were assessed. A representative immunoblot is shown (40 μg protein/lane; C, control; F, FGF21). (**B**) Eight-day-old mouse small intestine explants were treated with 10 nM FGF21 for 3 h, and transcript levels were assessed.

To assess the direct effects of FGF21 in the neonatal intestine, small intestine explants from 8-day-old pups were treated with FGF21 *in vitro*. Our results revealed that FGF21 rapidly and significantly induced the phosphorylation of ERK1/2, which is the canonical intracellular mediator of the intracellular effects of FGF21 (Fig. 5A). Moreover, exposure of neonatal small intestine explants to FGF21 significantly induced the expression of the intestinal hormone factor-encoding genes Gcg, Gip and Cck, as well as that of the gene encoding sucrase isomaltase (Sis) (Fig. 5B).

### Oral infusion of FGF21 to neonatal gut induces the gene expression of intestinal hormones and increases intestinal lactase activity and lactose absorption

**Figure 6:**
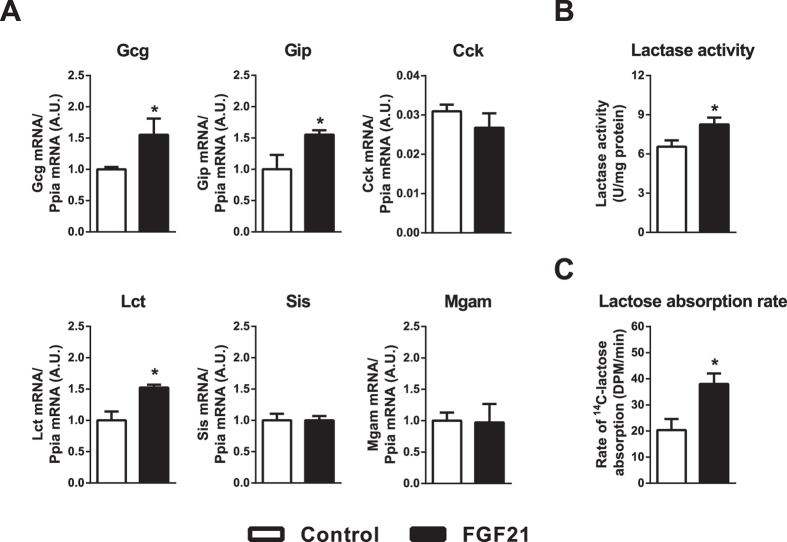
Oral infusion of FGF21 to neonatal gut induces the gene expression of intestinal hormones and increases intestinal lactase activity and lactose absorption. (**A**) Eight-day-old pups were subjected to intragastric infusion of 100 ng/ml FGF21 and after 4 h transcript levels were assessed. (**B**) Lactase activity was determined in the ileum from eight-day-old pups after 4 h of an intragastrically infusion with 100 ng/ml FGF21. (**C**) Lactase absorption rate was assessed by administering 14C-lactose in eight-day-old pups 4 h after 100 ng/ml FGF21 infusion and quantifying the breathed out radiolabeled CO_2_. Bars are means ± SEM of 4 independent experiments. Statistical significance of differences versus controls are shown as *P < 0.05, **P < 0.01.

To confirm these findings above about direct effects of FGF21 on neonatal intestine using an “*in vivo*” approach, we intragastrically infused to 8 day-old FGF21-KO pups with FGF21. Gene expression analysis in the small intestine revealed that FGF21 significantly induced the expression of Gcg, Gip and Lct genes (Fig. 6A). Moreover, lactase enzymatic activity was increased significantly by FGF21 gavage (Fig. 6B). To obtain a dynamic assessment of the effects of FGF21 on lactose absorption, we determined the effects of intragastrically infused FGF21 on the rate of 14C-lactose conversion to 14C-CO2 in neonatal mice. We found that FGF21 increased significantly the rate of lactose absorption and further metabolization (Fig. 6C). These findings confirmed the capacity of FGF21 to act directly in the lumen of the neonatal intestine and targeting intestinal hormones and digestive enzymes, and promoting lactose digestion.

## Discussion

In recent years, FGF21 has emerged as an important hormonal factor that has pleiotropic effects in metabolic regulation^9^. It is also important in neonatal life, as evidenced by the milk-intake-triggered induction of expression and FGF21 levels in newborn mice. High levels of FGF21 in neonatal mouse blood trigger important adaptations in the newborn, such as thermogenic activation of BAT^12^. The present study reveals that FGF21 is present in breast milk. This milk FGF21 does not contribute to systemic levels of FGF21 in mouse neonates; instead, it appears to act locally on the mouse neonatal intestine where it regulates the expression and activity of digestive enzymes and the synthesis and release of intestinal hormone factors, including the incretins GLP-1 and GIP1.

The presence of bioactive molecules in maternal milk has been increasingly recognized over the past few decades^1^. Milk proteins are resistant to degradation in the stomach; this is due to the low acidity of the infant stomach, and the protective (i.e. anti-proteolytic) environment that is formed by milk components as they enter the stomach^17^,^18^. Several growth factors are known to be transferred from maternal milk to the neonatal intestine, where they play important roles in promoting intestinal maturation. Adipokines, such as leptin and adiponectin, have also been found in maternal milk, and there is evidence that contribute to the systemic levels of these factors in neonates^4^,^19^. Here, we establish that FGF21 is among the endocrine factors that confer the important properties of breast milk and contribute to the maturation of the neonatal gastro-intestinal system, at least in the current rodent model. Maternal blood, rather than the mammary gland, appears to be the main source of milk FGF21; this is similar to previous findings for EGF^20^ and IGF-II^21^, but contrasts with those for leptin and adiponectin^13^,^22^. Consistent with a previous report^23^, we observed high blood FGF21 levels in lactating dams. This is likely to favor the substantial transfer of FGF21 to milk.

The small intestine expresses substantial levels of FGF receptors, mainly FGFR1, which can mediate responsiveness to FGFs. We found that β-Klotho, the co-receptor that mediates specific responsiveness to FGF21, is highly expressed in the luminal area of the neonatal (but not adult) small intestine. Due to this specific developmental regulated expression of β-Klotho, the neonatal gut becomes target of the effects of FGF21, as evidenced by the induction of ERK1/2 phosphorylation in the intestines of pups fed FGF21-containing milk and in FGF21-treated neonatal intestine explants “*in vitro*”.

A subset of the intestinal digestive enzymes, including lactase, sucrase isomerase and maltase glucoamylase, appears to be target of FGF21 in the neonatal intestine. However, the gene expression and activities of these enzymes were affected to different extent by our experimental manipulations to assess the effects of FGF21. In any case, dynamic assessment of the effects of FGF21 on lactose digestion and further metabolization as well as galatose levels revealed a positive action of FGF21 promoting lactose absorption. A second important set of genes that appear to be regulated by FGF21 in the neonatal intestine include the intestinal hormonal factors GLP-1 and GIP.

The systemic consequences of our experimental alterations in pups were moderate. FGF21-KO pups pups as well as WT pups nursed by FGF21-KO dams are viable, and body weight alterations are transient. However, the rescue of body weight reduction when FGF21-KO pups were fed by WT dams indicates that milk FGF21 may be relevant for optimal neonate development. The moderate, but significant, reduction in blood glucose and triglyceride levels associated with the lack of FGF21 in maternal milk is likely to reflect a mild alteration in the nutritional status of pups consistent with low body weight. Although non-detected alterations in the lactation performance of FGF21-KO dams, unrelated to the absence of FGF21 in milk, cannot be totally ruled out, the evidence of direct effects of FGF21 in neonatal intestine reported here, strongly suggest a role for milk FGF21. Perhaps, as in other gene-invalidation models, other factors present in milk could elicit homeostatic, compensatory, processes to moderate the impact of the lack of FGF21 in milk. Given the growing awareness of the impact of early nutritional and hormonal disturbances on later adult physiopathology both in rodents and humans^24^, additional research is warranted to examine whether inappropriate FGF21 signaling in the neonatal gut may contribute to later adult susceptibility to metabolic alterations. Although extrapolation of data from rodent models to humans should be done with caution, future studies should also examine the possible contribution of FGF21 signaling the neonatal gut to the optimal action of breast milk for enteral nutrition in highly premature infants with intestinal immaturity^25^.

**Figure 7:**
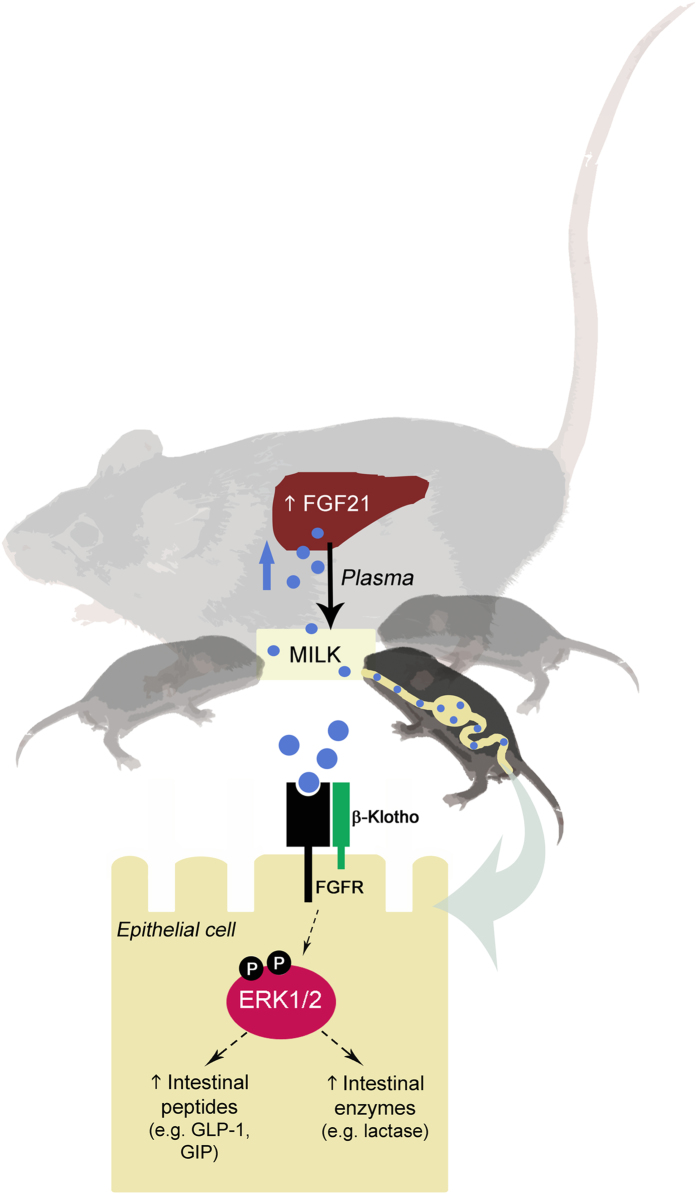
Summary of the action of maternal milk FGF21 on controlling the function of neonatal intestine. In lactating dams, lactation induces hepatic production of FGF21, which is transferred from plasma to milk and reaches neonatal intestine. FGF21 acts on the complex FGFR-β-Klotho present in the intestinal epithelial cells of neonates and promotes the production of intestinal peptides (e.g. GLP-1 or GIP) and intestinal digestive enzymes, such as lactase. *Mice photographs and drawings were performed by A. Gavaldà-Navarro.*

In summary, we herein report a novel biological role for FGF21 as a hormonal factor present in milk and involved in controlling neonatal intestine function (Fig. 7). Further studies will be required to confirm these findings in human neonates and therefore to establish their potential implications in the biomedical context of neonatal counseling, the importance of maternal breast feeding and the need to optimize biotechnological formula designs to fulfill the complexities of optimal milk composition and mimic the properties of breast milk.

## Methods

### Animals

Mice were maintained under standard conditions of light (12 h light/12 h dark cycle) and temperature (21 ± 1 °C); the care and use of mice was performed in accordance with the European Community Council Directive 86/609/EEC and all experimental procedures were approved by the Institutional Animal Care and Use Committee of the University of Barcelona.

### Acquisition and analysis of rodent milk

Milk samples from dams were collected by manual milking from anesthetized 15-day lactating mice and rats. FGF21 protein levels in milk and plasma were determined by ELISA (BioVendor). Milk analysis included measurement of triglycerides and lactose (both from Sigma-Aldrich), protein (Bio-Rad), individual fatty acid composition^26^, adiponectin (Invitrogen), leptin (Millipore).

### Measurements in human breast milk

Human FGF21 protein levels were measured in milk and serum (BioVendor) obtained from 4 (colostrum), 4 (transitional milk) and 14 (mature milk) healthy women between 27–37 years of age, after obtaining the informed consent from all subjects. For analysis of lactating mammary gland cell RNA, 5 milk samples (5 ml) were collected using a manual pump under aseptic conditions, stored at 4 °C, and processed within 6 hours for isolation of cells following a previously described method^27^ (See Supplemental Methods for further details). The studies were performed in accordance with the approved guidelines and procedures. The Bioethics Committee at the University of Barcelona, Spain, approved the human studies.

### ^125^I-FGF21 tracing

Fifteen days after parturition, lactating mice were intraperitoneally (i.p.) injected with 5 μCi of ^125^I-FGF21 (Phoenix Europe). Blood samples were obtained via direct puncture of the saphenous vein at 2, 8, 12 and 36 h after injection; milk was obtained at 36 h after injection. Neonatal plasma, stomach and total small intestine samples were obtained. The ^125^I-FGF21 was separated from free ^125^I by tricholoroacetic acid (TCA) protein precipitation, and radioactivity was assessed using a γ-counter (Packard Cobra II).

### Studies in neonatal mice and animal experimental designs

When indicated, we used FGF21-/- (FGF21-KO) mice (B6N;129S5-Fgf21tm1Lex/Mmcd, from the Mutant Mouse Regional Resource Centre, an NCRR-NIH-funded strain repository, donated to the MMRRC by Genentech, Inc) that had been backcrossed to the C57/BL6 background^28^.

For cross-feeding experiments, WT or FGF21-KO adult mice were mated. At the time of simultaneous deliveries (maximum variability in delivery time, 12 h) maximum), the following experimental groups were established: WT pups fed by WT dams, KO pups fed by KO dams; KO pups fed by WT dams, and WT pups fed by KO dams. All litters were adjusted to 7–8 pups and were fed by a non-progenitor dam, even for experiments in which the maternal and neonatal genotypes were the same. After 8 days of lactation, pups were killed by decapitation, blood was collected, plasma was obtained and tissues were collected and frozen for further processing. Blood glycemia (Accutrend^®^), plasma galactose (Abcam), NEFA and ketone bodies (Wako), triglycerides and glycerol (Sigma-Aldrich) were determined.

For oral infusion of FGF21, 8 day-old FGF21-KO pups were orally infused with 100 μl of 100 ng/ml FGF21 dissolved with 20% Intralipid™ (Fresenius Kabi) or 20% Intralipid™ alone (vehicle), using a 0,038” Intramedic^®^ polyethylene tube introduced into the stomach. After 4 h after administration, the pups were killed by decapitation, and tissues were collected and frozen for further processing.

### Intestinal explant incubation

Sections of jejunum (0.5-cm) from 8-day old pups were cut longitudinally and incubated with DMEM with or without 10 nM mouse recombinant FGF21 (BioVendor R&D). At the indicated times, tissue and media were collected for further analysis.

### RNA isolation, cDNA synthesis, and real-time PCR

Total RNA was isolated from rodent samples (tissues, scraped intestinal mucosa, and intestine explants), as well as from cells that had been isolated from human breast milk using a previously described method^27^. Independent samples of total RNA from human mammary gland were obtained from Clontech and AMS Biotechnology. RNA was prepared using a column-affinity based methodology (Macherey-Nagel). Total RNA (500 ng) was transcribed into cDNA using Multiscribe reverse transcriptase and random-hexamer primers (TaqMan Reverse Transcription Reagents; Applied Biosystems/Life Technologies). For quantitative analysis of mRNA expression, TaqMan quantitative real-time polymerase chain reaction (qPCR) was performed using a 7500 Real-Time PCR System (Applied Biosystems) and a final volume of 20 μl containing Platinum Quantitative PCR SuperMix-UDG with ROX reagent (Invitrogen) and specific primer pair/probe sets obtained from Applied Biosystems/Life Technologies. The relative mRNA expression levels of the different genes were normalized with respect to that of the 18S rRNA or Ppia mRNA (endogenous controls) using the comparative (2^−ΔCT^) method. Transcript levels were considered undetectable when the CT value was ≥40 under these experimental conditions.

### Western blot analysis

Tissue protein extracts, milk and plasma were analyzed by Western blotting using standard procedures. The utilized primary antibodies were directed against Phospho-p44/42 MAPK (P-Erk1/2) (Thr202/Tyr204) and p44/42 MAPK (Erk1/2) (both from Cell Signaling), β-Klotho (Abcam), FGF21 (Santa Cruz Biotechnology) and β-actin (Sigma-Aldrich). Immunoreactive signals were obtained using an enhanced chemiluminescence HRP substrate (Millipore).

### Immunohistochemical detection of β-Klotho

Intestinal segments were collected, fixed with 4% formalin for 24 h, and subsequently stored in ethanol 70% at 4 °C until paraffin infiltration was performed. Paraffin-embedded tissues were divided into transverse and longitudinal sections, and mounted on glass slides. Samples were incubated overnight at 4 °C with an anti-β-Klotho antibody (LifeSpan BioSciences), incubated with an ABC complex-conjugated secondary antibody, and treated with DAB for visualization of results. Finally, the samples were stained with hematoxylin 50x optical microphotographs were obtained.

### Quantification of intestinal peptides in plasma

The plasma concentrations of glucagon-like peptide 1 (GLP-1), gastric inhibitory peptide (GIP) and peptide YY (PYY) were determined using multiplex assays (Millipore Corporation).

### Lactase activity

Protein extracts were obtained from neonatal duodenum, jejunum and ileum, and lactase activity was assayed using a slight modification of the method described by Dahlqvist, A^29^.

### Lactose absorption rate measurement

Lactose absorption rate was assessed by quantifying the exhalation of ^14^CO_2_ produced from the oxidation of ^14^C-lactose infused to gut, following a previously described method^30^ adapted to mouse neonates. Briefly, after 4 hours of oral infusion of 100 ng/ml FGF21, pups were orally infused with 2.5 μC_i_^14^C-lactose (Hartmann Analytic) dissolved in 100 μl of water. Pups were maintained in a chamber which contained a 6 cm^2^ Whatman^®^ paper impregnated with phenylethylamine (Sigma). CO_2_ paper traps were collected every 30 minutes during 3 hours. CO_2_ paper traps were counted using a Packard 2100TR TriCarb Liquid Scintillation Counter and the linear rate of ^14^CO_2_ counts appearance was calculated.

### Statistical Analysis

All results are expressed as means ± SEM, and differences were compared using the unpaired t-test, one-way analysis of variance (ANOVA) with Tukey’s Multiple Comparison Test, or two-way ANOVA with Bonferroni post-test, as appropriate.

## Additional Information

**How to cite this article**: Gavaldà-Navarro, A. *et al.* Fibroblast growth factor 21 in breast milk controls neonatal intestine function. *Sci. Rep.* **5**, 13717; doi: 10.1038/srep13717 (2015).

## Supplementary Material

Supplementary Information
