# Supplementary material for: Fibroblast growth factor 21 in breast milk controls neonatal intestine function
Source: Sci Rep. 2015 Sep 2;5:13717. doi: 10.1038/srep13717 (PMC4557064; doi:10.1038/srep13717)
Supplement: Supplementary Information [file srep13717-s1.pdf]

Fibroblast growth factor 21 in breast milk controls neonatal intestine function

Aleix Gavaldà-Navarro[1, 2], Elayne Hondares[1, 2], Marta Giralt[1, 2], Teresa Mampel[1, 2], Roser Iglesias[1, 2], Francesc Villarroya[1, 2]

[1]Departament de Bioquimica i Biologia Molecular, Institute of Biomedicine (IBUB), University of Barcelona, and [2]CIBER Fisiopatologia de la Obesidad y Nutrición, Av Diagonal 643, 08028 Barcelona, Catalonia, Spain.

Supplementary Information contains four Supplemental Figures, four Supplemental Tables and detailed description of Methods.

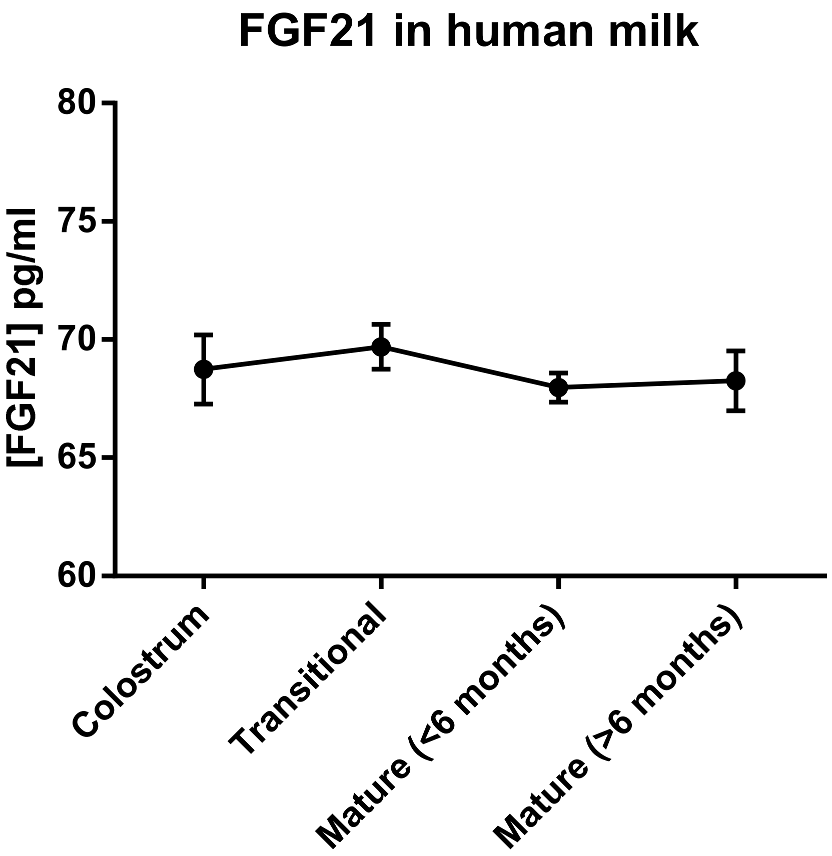

Supplemental Figure 1. FGF21 content in human milk at different stages of lactation; Related to Figure 1. Data are means ± SEM of 3 samples for colostrom and transitional milk, 10 samples for mature milk under 6 months and 4 samples for mature milk over 6 months.

Supplemental Table 1. FGF21 expression in liver, brown adipose tissue (BAT) and mammary gland from non-pregnant, non-lactating females (control) and dams at days 4, 8, 15 and 21 of lactation; Related to Figure 1. Data are Ct means of FGF21 mRNA (FGF21) and 18S rRNA (18S) RT-PCR amplification of RNA in the conditions described in the methods section (4-6 independent control females or lactating dams per group). Ct >40 are considered undetectable.

	Control females		Lactating day 4		Lactating day 8		Lactating day 15		Lactating day 21	
	FGF21	18S	FGF21	18S	FGF21	18S	FGF21	18S	FGF21	18S
Liver	33,2	12,6	32,2	12,4	28,4	12,5	29,71	12,9	34,0	12,8
BAT	32,8	13,1	36,1	13,0	34,2	12,9	35,9	12,9	35,9	13,4
Mammary gland	>40	13,2	>40	12,4	>40	12,1	>40	11,9	>40	12,3

Supplemental Table 2. FGF21 expression in human liver, resting mammary gland and lactating mammary gland; Related to Figure 1. Data are Ct means of FGF21 mRNA (FGF21) and 18S rRNA (18S) RT-PCR amplification of RNA in the conditions described in the methods section (2-5 independent samples). Ct >40 are considered undetectable.

	FGF21	18S
**Liver (Biopsies)**	32,4	13,7
**Resting mammary gland (Biopsies)**	>40	14,3
**Lactating mammary gland (Cells in milk)**	>40	14,1

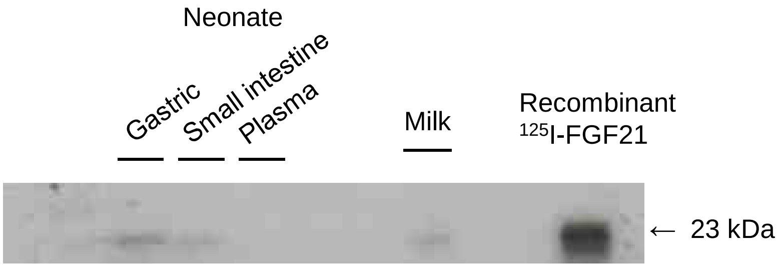

Supplemental Figure 2. Autoradiography of dam's milk, gastric and intestinal contents 36h after injection of 15 day-lactating mice with 5 μCi of 125I-FGF21; Related to Figure 1. Autoradiography of dam's milk, gastric and intestinal content 36h after injection of 5 μCi of 125I-FGF21 to 15 day-lactating mice. Lanes show autoradiography of SDS-PAGE electrophoresis of 15 μl neonatal stomach content (gastric), 50 μl of intestine homogenate (1:3 w/v) and 40 μl neonatal plasma, 40 μl milk.

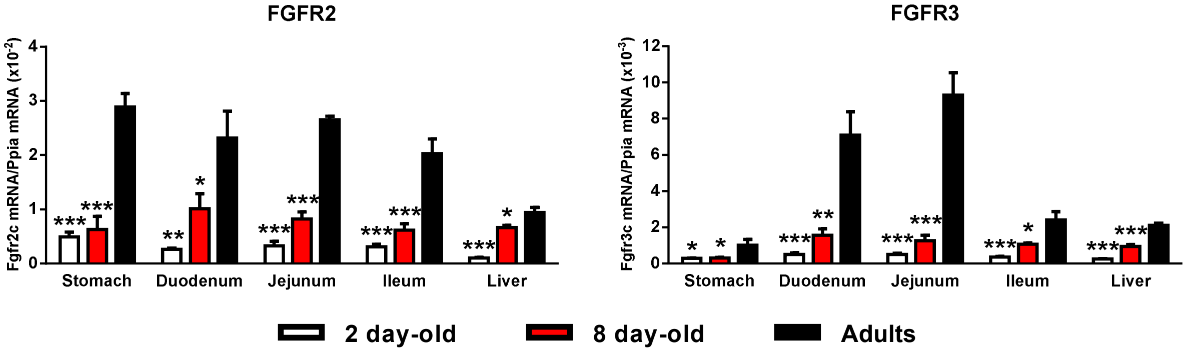

Supplemental Figure 3. FGFR2 mRNA and FGFR3 mRNA expression levels in the stomach, duodenum, jejunum, ileum and liver of 2-day-old pups, 8-day-old pups and adults; Related to Figure 2. Data are means ± SEM of samples pooled from 4-6 independent litters. Statistically significant changes relative to adults are shown as *P<0.05, ** P<0.01 and *** P<0.001.

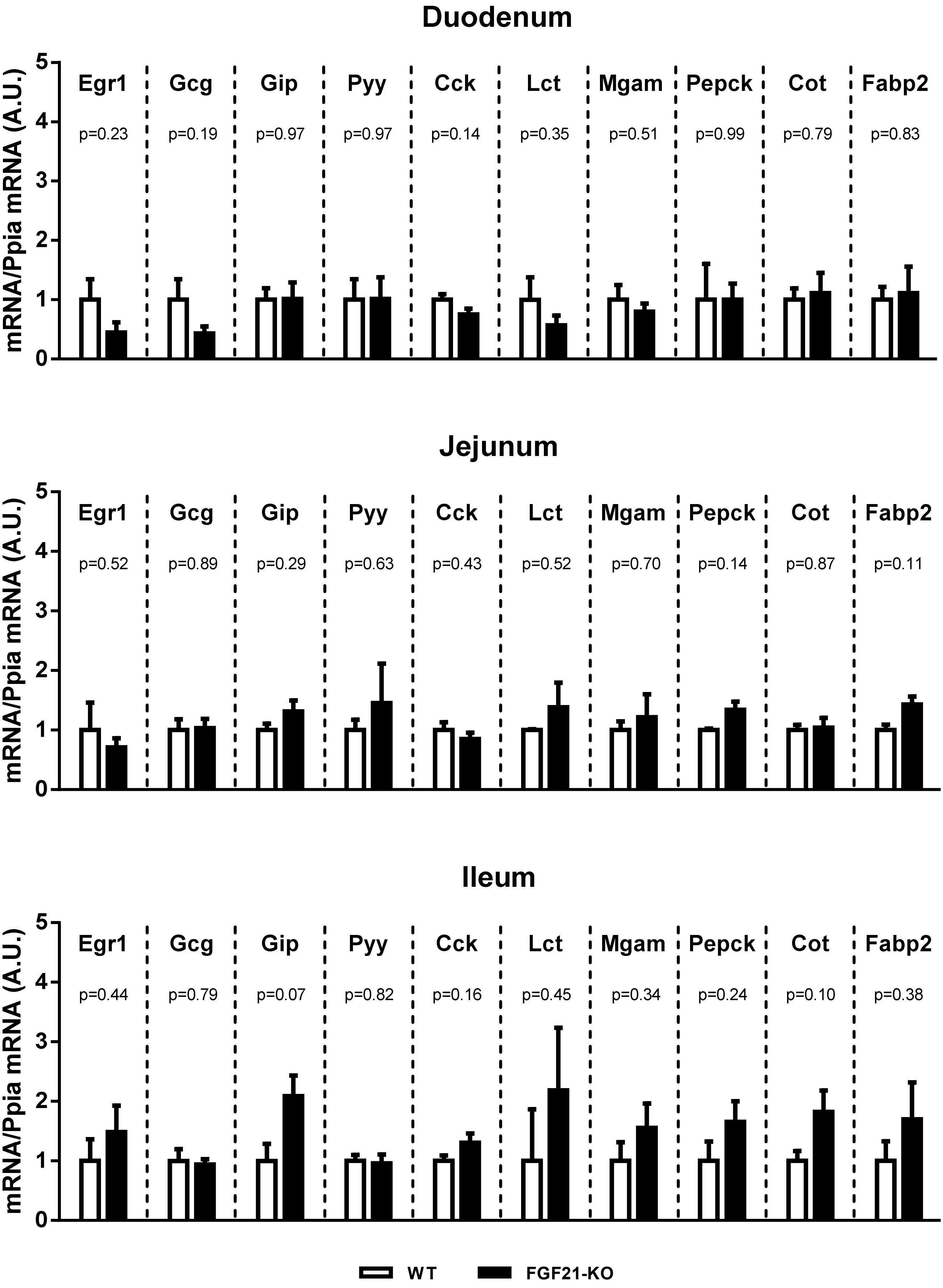

Supplemental Figure 4. Gene expression in small intestine from adult FGF21-KO and WT mice; Related to Figure 3. Data are means ± SEM of 5-6 independent assays from 5-month-old male FGF21-KO mice and WT controls, that had been fed by FGF21-KO dams or WT dams, respectively. P values for statistical comparison are shown.

Supplemental Table 3. Milk compositions of WT and FGF21-KO lactating dams; Related to Figure 3. Data are means ± SEM of milk obtained at day 15 of lactation from 5 independent dams per group.

	Wild-type	***FGF21-KO***	***P-value***
Lactose (mM)	78.58 ± 4.36	77.06 ± 16.12	0.88
Protein (mg/dl)	2113 ± 245	2235 ± 362	0.65
Triglycerides (mg/dl)	2393 ± 748	2240 ± 717	0.81
Lipid composition (% of total)			
Saturated fatty acids (SFA)	44.05 ± 0.90	44.90 ± 4.13	0.75
Monounsaturated fatty acids (MUFA)	29.12 ± 1.89	28.93 ± 3.72	0.94
Polyunsaturated fatty acids (PUFA)	26.82 ± 1.02	26.17 ± 0.91	0.46
ω-3 PUFA	1.29 ± 0.07	1.36 ± 0.10	0.41
ω-6 PUFA	25.53 ± 0.96	24.81 ± 0.82	0.38
Adiponectin (µg/ml)	2.02 ± 1.01	0.76 ± 0.65	0.09
Leptin (pg/ml)	3789 ± 1391	2443 ± 370	0.32

Supplemental Table 4. Metabolic profile of FGF21-KO neonates fed by FGF21-KO dams or WT dams and WT pups fed by FGF21-KO dams, compared to WT neonates fed by WT dams. Data are means ± SEM of 5-6 independent assays from to 8-day-old neonates fed under the indicated conditions since day 1 after birth. Statistically significant changes relative to WT pups fed by WT dams are shown as [a]P<0.05. P values from comparison between factors, calculated by two-factor ANOVA, are also shown.

					Comparison between factors (P values)	
	WT pups fed by WT dams	***KO pups fed by WT dams***	***KO pups fed by KO dams***	***WT pups fed by KO dams***	***Pup's genotype***	***Nursing dam's genotype***
Glucose (mg/dl)	150.5 ± 6.84	139.5 ± 4.32	131.7 ± 7.89	133.4 ± 5.15	0.302	**0.046**
Triglycerides (mg/dl)	8.23 ± 0.46	7.91 ± 0.54	7.35 ± 0.51	6.10 ± 0.34[a]	0.321	**0.005**
Glycerol (mg/dl)	6.33 ± 0.55	4.97 ± 0.50	5.93 ± 0.81	4.11 ± 0.28[a]	0.686	0.266
Non-esterified fatty acids (mg/dl)	36.56 ± 2.29	36.56 ± 2.88	36.85 ± 3.49	29.74 ± 1.46	0.156	0.191
3-Hydroxybutyric acid (mM)	0.98 ± 0.04	0.95 ± 0.07	0.99 ± 0.07	0.75 ± 0.05[a]	0.071	0.128

Supplemental Methods

Animal experimental designs

Mice were maintained under standard conditions of light (12h light/12h dark cycle) and temperature (21±1°C). The care and use of mice was in accordance with the European Community Council Directive 86/609/EEC and all experimental procedures were approved by the Institutional Animal Care and Use Committee of the University of Barcelona.

Acquisition and analysis of rodent milk

To study the concentration of FGF21 in milk, adult female Swiss mice and Wistar rats were mated with appropriate males. After delivery, all litters were adjusted to 9-10 pups. Milk samples were collected from dams on day 15 of lactation. Nursing dams were separated from their pups for 12 h (to ensure that the mammary glands were full of milk), anesthetized with isoflurane and manually milked. Milk samples were stored frozen at -20ºC. For analysis, each sample was centrifuged (13000 rpm for 10 min at 4ºC) for removal of the fat layer. Blood samples were collected from the end of the tail. The FGF21 protein levels in milk and plasma from mice and rats were determined by ELISA (RD291108200R, BioVendor R&D, Brno – Modřice, Czech Republic).

The milk composition was determined as follows. Triglycerides were measured using a commercial kit (Sigma-Aldrich). The protein content was measured by Bradford method (Bio-Rad Protein Assay; Bio-Rad Laboratories GmbH, Munich, Germany). The lactose content was spectrophotometrically quantified based on the

production of NAD by the coupled reactions catalysed by β-galactosidase and glucose/hexokinase-glucose 6-phosphate dehydrogenase (Glucose Assay Reagent) (Sigma-Aldrich, St. Louis, MO). The individual fatty acid composition was determined by gas chromatography using a gas chromatograph/mass spectrometer (Shimadzu GCMS-QP2010 Plus) and a reference FAME mixture (GLC-744 Nu-Chek Prep), essentially following the method described by Lepage (26). Adiponectin levels were measured by ELISA (KMP0041; Invitrogen; Life Technologies, Foster City, CA). The levels of leptin were determined by a MILLIPLEX® MAP Mouse Adipokine Assay (Cat. No. MADKMAG-71K, Millipore Corporation, Billerica, MA).

Measurement of FGF21 in human breast milk

Mature breast milk (sampled at least 1 month after parturition) and blood samples were obtained from donors who voluntarily consented to participate in the study. The study subjects comprised 22 healthy women between 27 and 37 years of age. According to the time of lactation, milk samples were divided in colostrum (day 1 to 5 after birth) (n=4), transitional milk (day 6 to 21) (n=4), fully mature milk under 6 months (n=10) and fully mature milk over 6 months (n=4). For analysis of FGF21 analysis, skim milk was prepared by centrifugation of whole milk at 13000 rpm for 10 min at 4ºC. Human FGF21 protein levels in milk and serum were determined by ELISA (RD191108200R, BioVendor R&D). This portion of the study was approved by Bioethics Committee at the University of Barcelona, Spain.

Expression analysis in human mammary gland

Two commercial resting mammary gland RNA samples were obtained from a 27-year-old sudden dead Caucasian female (Clontech Laboratories, Inc.; Ref. 636576) and from a single healthy normal donor (AMS Biotechnology; Ref. HR-414). Lactating mammary gland RNA was obtained from cells present in 5 fresh milk samples, following a previously described method (28). Milk samples were collected using a manual pump under aseptic conditions, stored at 4 ºC, and processed within 6 hours after expression. Briefly, milk samples were diluted with PBS 1x (1:1), centrifuged at 800 xg for 20 min at room temperature, and upper fat layer and serum phase were removed. Cell pellets were washed twice with PBS 1x and RNA was purified as described in the below section "RNA isolation, cDNA synthesis, and real-time PCR". Three liver samples obtained from biopsies were used as positive controls of FGF21 expression. In both cases, informed consent from all subjects were obtained and the study was approved by the Bioethics Committee at the University of Barcelona, Spain.

Studies in lactating mice

Lactating dams (days 4, 8, 15 and 21 after delivery) and virgin female mice of the same age (controls) were sacrificed by decapitation. Blood was collected, plasma was obtained, and mammary gland, liver, periovaric WAT and interscapular BAT were dissected and frozen in liquid nitrogen for further analysis.

^{125}I-FGF21 tracing

Lactating dams (15 days after parturition) were injected intraperitoneally with 5 μCi of ^{125}I-FGF21 (Phoenix Europe GmbH, Karlsruhe, Germany) dissolved in 500 μl of saline, and then kept with their pups during 36 h. Blood samples were obtained via direct puncture of the saphenous vein at 2, 8, 12 and 36h after injection. Thirty-six hours after injection, the lactating dams were anesthetized, and milk was obtained as described above. Pups were sacrificed, plasma samples were obtained, and the stomach and total small intestine were removed, weighed and mechanically homogenized in 2 ml of 1x PBS. Proteins from plasma, milk, stomach and small intestine were precipitated by addition of 1:10 volume of 10% tricholoroacetic acid (TCA). Centrifugation was used to separate precipitated proteins containing ^{125}I-FGF21 from free ^{125}I (in the supernatant). Radioactivity was counted from the precipitate and supernatant (to confirm efficient separation) using a γ-counter (Packard Cobra II).

Studies in neonatal mice and animal experimental designs

For studies in neonatal development, C57/BL6 pups were studied 2, 8 and/or 15 days after birth. When indicated, we used FGF21-/- (FGF21-KO) mice (B6N;129S5-Fgf21tm1Lex/Mmcd; obtained from the Mutant Mouse Regional Resource Centre, an NCRR-NIH-funded strain repository, and donated to the MMRRC by Genentech) that had been backcrossed to C57/BL6 background (27). Pups were killed by decapitation; blood was collected for preparation of serum; and tissues were immediately frozen in liquid nitrogen, and stored at -80ºC until

processing. For comparison, tissues were also obtained from adult mice (male, 5 month-old).

To study the specific localization of β-Klotho expression in the intestinal mucosa, small intestines were removed from 15 day-old lactating mice. Samples of the duodenum, jejunum and ileum were cut longitudinally to expose the luminal areas, and a blade was used to scrape the mucosa and separate it from the rest of the tissue, following the previously reported procedure (15). Unprocessed tissue (total), mucosa and tissue remaining after scraping were frozen for RNA isolation and gene expression analysis.

For cross-feeding experiments, WT or FGF21-KO adult mice were simultaneously mated with the appropriate males, and the presence of vaginal plugs (indicating pregnancy) was monitored in order to obtain simultaneous WT and KO deliveries. At birth, the litter sizes were adjusted to 7 or 8 individuals and pups were interchanged with dams; a maximum 12-h variability in delivery time was allowed. The experimental groups consisted of WT pups fed by WT dams, KO pups fed by KO dams and KO pups fed by WT dams and WT pups fed by KO dams. All litters were fed by a non-progenitor dam even when maternal and neonatal genotypes were the same. After 8 days of lactation, pups were killed by decapitation, blood was collected, plasma was obtained, and tissues were collected and frozen for further processing. Blood glycemia was determined using Accutrend® (Roche). Commercially available kits were used to assess plasma galactose (ab83382; Abcam), insulin (RSHAKRIN031R; BioVendor R&D), NEFA (434-91795, 436-91995; Wako Chemicals GmbH, Neuss, Germany), ketone

bodies (417-73501, 413-73601; Wako), triglycerides (TR0100; Sigma-Aldrich) and glycerol (FG0100; Sigma-Aldrich).

For oral infusion of FGF21, 8-day-old FGF21-KO pups were separated from dams 4 h, and then infused with 100 μl of FGF21 100 ng/ml dissolved with 20% *Intralipid*™ (Fresenius Kabi, Bad Homburg, Germany) or 20% *Intralipid*™ alone (vehicle). For infusions, 0,038” Intramedic® polyethylene tubing (Clay Adams, Becton, Dickinson and Company, Franklin Lakes, NJ) was introduced into the stomach and coupled to a 1-ml syringe, as previously described (Hondares et al. 2010). The use of *Intralipid*™ facilitated *de visu* gastrointestinal tracing. Four hours after administration, pups were killed by decapitation and tissues were collected and frozen for further processing.

Intestinal explant incubation

Jejunum samples were collected from 8 day-old pups, divided into 0.5 cm sections, cut longitudinally to expose the mucosa, and incubated with DMEM with or without 10 nM mouse recombinant FGF21 (RD272108100, BioVendor R&D) at 37 °C in a humidified 95% air/5% CO_2 incubator. At the indicated times (10 min, 20 min and 3h), tissue and media samples were collected together and centrifuged, and pellets were frozen for subsequent RNA isolation (for gene expression studies) or protein homogenization (for Western blot analysis).

RNA isolation, cDNA synthesis, and real-time PCR

Dissected tissues were homogenized using an IKA® T25 digital ULTRA-TURRAX (Staufen, Germany), and total RNA was isolated using a column-affinity based methodology (NucleoSpin RNA II; Macherey-Nagel, Düren, Germany). Total RNA was isolated from rodent samples (tissues, scraped intestinal mucosa, and intestine explants), as well as from cells that had been isolated from human breastmilk using a previously described method (28). Independent samples of total RNA from human mammary gland were obtained from Clontech and AMS Biotechnology. Total RNA (500 ng) was transcribed to cDNA using Multiscribe reverse transcriptase and random-hexamer primers (TaqMan Reverse Transcription Reagents; Applied Biosystems/Life Technologies, Foster City, CA). For quantitative analysis of mRNA expression, TaqMan quantitative real-time polymerase chain reaction (qPCR) was performed on a 7500 Real-Time PCR System (Applied Biosystems), a final volume of 20 µl containing Platinum Quantitative PCR SuperMix-UDG with ROX reagent (Invitrogen), and the following specific primer pair/probe sets: Fgfr1 (Fibroblast growth factor receptor 1), Mm00438930_m1; Fgfr2 (Fibroblast growth factor receptor 2), Mm01269937_m1; Fgfr3 (Fibroblast growth factor receptor 3), Mm01216080_m1; Fgfr4 (Fibroblast growth factor receptor 4), Mm00433314_m1; Klb (Klotho beta), Mm00473122_m1; Cdh1 (Cadherin 1), Mm01247357_m1; Acta2 (Actin α2), Mm00725412_s1; Pck1 (Phosphoenolpyruvate carboxykinase 1), Mm00440636_m1; Hmgcs2 (3-hydroxy-3-methylglutaryl-coenzyme A synthase 2), Mm00550050_m1; Crot (Carnitine O-octanoltransferase), Mm00470079_m1; Cpt1a (Carnitine palmitoyltransferase 1a), Mm01231183_m1; Fabp2 (Fatty acid binding protein 2), Mm00433188_m1;

Ppargc1a (Peroxisome proliferative activated receptor, gamma, coactivator 1 alpha; PGC-1α), Mm00447183_m1; Slc2a1 (Solute carrier family 2, member 1; Glut1), Mm00441480_m1; Lct (Lactase), Mm01285112_m1; Mgam (Maltase-glucoamylase), Mm01163791_m1; Sis (Sucrase isomaltase), Mm01210305_m1; Gcg (Glucagon), Mm01269055_m1; Gip (Gastric inhibitory polypeptide), Mm00433601_m1; Cck (Cholecystokinin), Mm00446170_m1; Pyy (Peptide YY), Mm00520716_g1; 18S rRNA, Hs99999901 and Ppia (Peptidylprolyl isomerase A), Mm02342430_g1 (TaqMan Gene Expression Assays; Applied Biosystems). The relative mRNA expression levels of the different genes were normalized with respect to that of the 18S rRNA or Ppia mRNA (endogenous controls) using the comparative ($2^{-\Delta CT}$) method. Transcript levels were considered undetectable when the CT value was >40 under our experimental conditions.

Western blot analysis

Dissected tissues were homogenized using an IKA® T25 digital ULTRA-TURRAX (Staufen, Germany) in lysis buffer (250 mM saccharose, 20 mM Na-HEPES pH 7.4, 10 mM KCl, 1.5 mM $MgCl_2$, 1 mM EDTA, 1 mM EGTA, 40 mM β-glycerophosphate, 2 mM Na_3VO_4, 1 mM phenylmethylsulfonyl fluoride [PMSF], 1 mM dithiothreitol [DTT], and a complete protease inhibitor cocktail (Roche Applied Science). Lysates were centrifuged at 1,500 ×g for 5 minutes at 4 °C to remove intact cells, and the proteins were quantified using the Bradford method. For Western blotting, proteins (40 μg) were heated for 5 minutes at 95 °C under reducing conditions and then resolved by 12% sodium dodecyl sulfate-

polyacrylamide gel electrophoresis (SDS-PAGE) for 3 h at a constant voltage of 100 mV. The resolved proteins were transferred electrophoretically to PVDF (polyvinylidene difluoride) membranes (Immobilon; Millipore, Billerica, MA) for 1 h at a constant amperage of 400 mA, and then blocked in 5% (wt/vol) nonfat milk in 1x PBS/1% Tween-20 (Sigma-Aldrich) for 1 hour at room temperature with agitation. Each membrane was exposed to primary anti-Phospho-p44/42 MAPK (P-Erk1/2) (Thr202/Tyr204) (#9101, Cell Signaling Technology Inc., Danvers, MA), anti-p44/42 MAPK (Erk1/2) (#9102, Cell Signaling), anti-β-Klotho (ab76356, Abcam Plc, Cambridge, UK) or anti-β-actin (Sigma-Aldrich) antibodies, diluted in 1x PBS/0.1% Tween-20, overnight at 4 °C with agitation. Milk and plasma samples from human, mouse and rat were resolved in 13% SDS-PAGE, transferred to Immobilon-P membranes (Millipore) and probed with and antibody against FGF21 (V16) (sc-16842, Santa Cruz Biotechnology Inc., Santa Cruz, CA). Membranes were incubated with a horseradish peroxidase (HRP)-coupled secondary antibody (in 1x PBS/0.1% Tween-20) for 1 h at room temperature with agitation. Signals were detected with enhanced chemiluminescence HRP substrate (Millipore) and analysed with a Luminescent Image Analyzer LAS-3000 (Fujifilm Life Science, Tokyo, Japan). Signal intensities were quantified using Multi Gauge software (Fujifilm).

Immunohistochemical detection of β-Klotho

During tissue collection, intestinal segments were fixed with 4% formalin for 24 h and then stored in ethanol 70% at 4 ºC until paraffin infiltration was performed. Paraffin-embedded tissues were sectioned to obtain transverse and longitudinal

orientations, and the sections were mounted on glass slides. The samples were incubated with an anti-β-Klotho antibody (LS-B3568, LSBio, LifeSpan BioSciences Inc., Seattle, WA) in a humidified chamber overnight at 4 ºC and then incubated with an ABC-complex-conjugated secondary antibody, and the results were visualized with DAB. Finally, samples were stained with hematoxylin and photographed under a microscope (50x magnification).

Quantification of intestinal peptides in plasma

The concentrations of glucagon-like peptide 1 (GLP-1), gastric inhibitory peptide (GIP) and peptide YY (PYY) in plasma samples from the various pups were determined by MILLIPLEX® MAP Mouse Metabolic Disease Multiplex Assay (Cat. No. MMHMAG-44K, Millipore).

Lactase activity

Lactase activity was assessed with a slight modification of the method reported by Dahlqvist (29). Approximately 25 mg of duodenum, jejunum or ileum were homogenized in 6 volumes of NaCl 0,9%. The homogenates were incubated with 4 volumes of 150 mM lactose in 0,1 M maleate buffer (pH 6,5) supplemented with 0,2 mM of the protease inhibitor, 4-chloromercuribenzoic acid, for 20 minutes at 37 ºC. The reaction was stopped with 6% perchloric acid, and samples were centrifuged at maximal speed. The pellet was removed, the supernatant was neutralized with 30% KOH, and the glucose concentration was determined with the Glucose Assay Reagent (Sigma-Aldrich). Enzymatic activity was normalized with respect to the quantity of input protein.

Lactase absorption rate measurement

Lactase absorption rate was assessed by quantifying the exhalation of $^{14}CO_2$ produced from the oxidation of ^{14}C-lactose infused to gut, following a previously described method (30) adapted to mouse neonates. Briefly, after 4 hours of oral infusion of 100 ng/ml FGF21 (described above in *Studies in neonatal mice and animal experimental designs*), pups were infused with 2.5 μC_i ^{14}C-lactose (MC-1466; Hartmann Analytic GmbH, Braunschweig, Germany) dissolved in 100 µl of water and maintained in a chamber which contained a 6 cm^2 Whatman® paper (GE Healthcare, Little Chalfont, UK) impregnated with phenylethylamine (Sigma), in order to capture the radiolabeled CO_2 breathed by the pups after lactose oxidation. Papers were changed every 15 minutes during the first half hour after ^{14}C-lactose administration and every 30 minutes during the following 2.5 hours, in order to obtain serial quantifications. CO_2 paper traps were placed in scintillation vials containing 5 ml of scintillation fluid (Ecoscint™ H, National Diagnostics, Atlanta, GA) and the samples were counted using a Packard 2100TR TriCarb Liquid Scintillation Counter (Packard Instrument Company Inc., Meriden, CT). The linear rate of $^{14}CO_2$ counts appearance was calculated.

Statistics

All results are expressed as means ± SEM. Differences were tested for statistical significance using unpaired t-test, one-way analysis of variance (ANOVA) with Tukey's Multiple Comparison Test, or two-way ANOVA with Bonferroni post-test, as appropriate.
